# Effects of UBE3A on the insulin resistance in polycystic ovary syndrome through the ubiquitination of AMPK

**DOI:** 10.1186/s12902-023-01400-8

**Published:** 2023-07-17

**Authors:** Ning Ma, Jing Zhou, Zhi Zhou, Bangbei Wan, Weiying Lu

**Affiliations:** Reproductive Medical Center, Hainan Women and Children’s Medical Center, No.75 Longkun South Road, Haikou, Hainan, 570206 China

**Keywords:** Ubiquitin-protein ligase E3A, Polycystic ovary syndrome, Denosine 5‘-monophosphate (AMP)-activated protein kinase, Insulin resistance

## Abstract

**Background:**

Polycystic ovary syndrome (PCOS) is a reproductive hormonal abnormality and a metabolic disorder, which is frequently associated with insulin resistance (IR). We aim to investigate the potential therapeutic effects of Ubiquitin-protein ligase E3A (UBE3A) on IR in the PCOS rats via Adenosine 5‘-monophosphate (AMP)-activated protein kinase (AMPK) activation.

**Methods:**

The PCOS and IR rats model was established by dehydroepiandrosterone (DHEA) and high fat diet (HFD) treatment, and the fat rate, glucose tolerance and insulin tolerance were measured. The IR rats numbers were calculated. Besides, the mRNA levels of glucose transporter 4 (GLUT4) and UBE3A were detected by RT-qPCR. Furthermore, the relationship between was demonstrated by co-IP assay. The phosphorylation and ubiquitination of AMPK were analyzed by western blot.

**Results:**

UBE3A was up-regulated in the PCOS rats. UBE3A knockdown significantly decreased the fat rate, glucose tolerance and insulin tolerance in the PCOS and IR rats. Additionally, the GLUT4 levels were significantly increased in PCOS + IR rats. Besides, after UBE3A knockdown, the IR rats were decreased, the p-IRS1 and p-AKT levels were significantly up-regulated. Furthermore, UBE3A knockdown enhanced phosphorylation of AMPK through decreasing the ubiquitination of AMPK. AMPK knockdown reversed the role of UBE3A knockdown in the PCOS + IR rats.

**Conclusions:**

UBE3A knockdown inhibited the IR in PCOS rats through targeting AMPK. Our study indicated that UBE3A might become a potential biological target for the clinical treatment of PCOS.

**Supplementary Information:**

The online version contains supplementary material available at 10.1186/s12902-023-01400-8.

## Introduction

Polycystic ovarian syndrome (PCOS) is an endocrine disorder syndrome characterized by persistent anovulation, hyperandrogenism and insulin resistance [1]. PCOS usually occurs adolescence and is the main cause of infertility in women [2]. In recent years, the incidence rate of PCOS increases yearly, which is one of the common diseases seriously endangering women’s physical and mental health [3]. The pathogenesis of PCOS is complex. Studies have found that insulin resistance (IR) and compensatory hyperinsulinemia play an important role in the occurrence and development of PCOS, leading to the continuous aggravation of the PCOS patient’s condition [4, 5]. On the one hand, high insulin level directly acts on ovarian thecal cells through insulin receptor, causing excessive functional androgens [6]; On the other hand, high insulin level disrupts the normal function of the hypothalamus-pituitary-ovary gonadal axis, which ultimately cause infertility [7]. Previous studies found that most PCOS patients have different degrees of IR, and the androgen level and insulin level are positively correlated [8, 9]. Therefore, reducing IR may be a promising therapeutic strategy for clinical treatment of PCOS.

Ubiquitin-protein ligase E3A (UBE3A), an important member of E3 ubiquitin ligase, is located on human chromosome 15q11-13, also known as human papillomavirus E6-associated protein (E6-AP) [10]. The molecular weight of UBE3A is about 100 kDa and UBE3A is composed of 865 amino acid residues [11]. As for the research on the role of UBE3A in the occurrence and development of human diseases, it is well known that UBE3A deletion will lead to the occurrence of Angelman syndrome [12]. Then, a large number of studies have shown that UBE3A is abnormally expressed in many cancer cells, which further affects various malignant biological behaviors of tumors, such as cervical cancer [13], liver cancer [14], lung cancer [15] and skin cancer [16]. It is found that UBE3A modifies ubiquitination of the target protein by connecting ubiquitin to a specific site, and then the target protein is degraded by the proteasome complex. When UBE3A is abnormally expressed, the target protein will accumulate abnormally (low levels of UBE3A) or degrade excessively (high levels of UBE3A function), which will eventually cause the occurrence and development of the disease [17–19]. Here, we found that UBE3A was significantly up-regulated in the PCOS patients through the bioinformatic analysis. However, the role of UBE3A in PCOS remains unclear.

Therefore, in this study, we systematically explored the effects of UBE3A on IR in PCOS. We hypothesized that UBE3A might degrade the Adenosine 5‘-monophosphate (AMP)-activated protein kinase (AMPK) to modulate the PCOS progression. Thus, UBE3A might act as an inducible protein in PCOS.

## Materials and methods

### Cell culture

The KGN cells and HEK 293T cells (ATCC, USA) were cutured in DMEM/F12 medium in a humidified incubator at 37° C with 5% CO2. The medium was supplemented with 10% FBS and 100 U/mL penicillin-streptomycin (Invitrogen).

### Quantitative real-time PCR (qPCR)

Total RNA was isolated using the TRNzol Universal reagent (TianGen, Beijing). Reverse transcription and qPCR were conducted using a one-step RT-QPCR kit (SYBR Green) (KeyGEN). qPCR was performed on a Line-Gene Real Time PCR system (Bioer, Hangzhou, China). The relative expression was analyzed using 2^−ΔΔCT^ method [20]. GAPDH was selected as the normalization.

### Animals

Forty-two female Sprague Dawley rats (6 weeks old, 200 ± 20 g) were obtained from the Shanghai Laboratory Animal Centre (SLAC, Shanghai, China), and were adaptively fed for 3 days on a 12 h light/dark cycle with free access to water (22 ± 1 ℃, 50–60% humidity ). The rats were first randomly divided into the following seven groups (n = 6 per group): control, PCOS, PCOS-high fat diet (IR), IR + Lentivirus (Len)-sh-nc, IR + Len-sh-UBE3A, IR + Len-sh-UBE3A + Len-sh-nc, and IR + Len-sh-UBE3A + Len-sh-AMPK. For PCOS model establishment, dehydroepiandrosterone (DHEA, Sigma) was diluted in 200 µL sesame oil, and the rats were injected with DHEA at a dose of 60 mg/kg body weight every day for 21 d. For IR induction, the POCS rats were fed with high-fat diet (HFD) at same time. The rats in the control group received a standard diet. The HFD contained the following ingredients: crude protein: 12.7%, crude fat 18.9%, crude fiber 3.8%, crude ash 4.0%, moisture 9.2%, calcium 0.82%, total phosphorus 0.69%, nitrogen-free extract 53.1%, total energy 4.34 kCal/g, energy supply ratio: protein 11.7%, fat 39.3%, carbohydrate 49%. Additionally, for UBE3A and AMPK knockdown, the rats were injected with Len-sh-nc, Len-sh-UBE3A, Len-sh-AMPK (1 × 10^8^ PFU, 10 µL ) one week before modeling. Finally, according to the homeostasis model assessment of IR (HOMA-IR) method, HOMA-IR = FINS × FPG/22.5. HOMA-IR > 2.8 indicated the successful induction of IR. The fat rate of rat in each group was detected using EchoMRI 100 (Echo Medical Systems, US).

### Sample collection

The weights and HOMA-IR of all rats in the each groups were determined after the experiment. Then, all rats were were euthanized using 1% pentobarbital sodium (150 mg/kg) to collect the blood from the abdominal aorta and ovary tissue samples. The samples were stored at − 80 ℃ for subsequent experiments.

### Glucose tolerance test (GTT)

At 6 and 12 weeks after experiments, the rats were fasted. After 12 h of fasting, the weight and fasting blood glucose level of rats in each group were measured. According to the weight of mice, 20% glucose solution (2 g/kg) was injected intraperitoneally. The blood glucose levels of rats in each group were measured at 30, 60, 90 and 120 min after injection. Finally, the area under curve (AUC) was calculated according to the blood glucose change at different measurement time, and the glucose tolerance of mice in each group was tested according to a previous study [21].

### Insulin tolerance test (ITT)

At 6 and 12 weeks after experiments, the rats were fasted. After 4 to 6 h of fasting, the weight and fasting blood glucose level of rat in each group were measured. Then the 0.75 U/kg insulin solution was injected intraperitoneally. At 15, 30, 45 and 60 min after the injection of insulin, the blood glucose level of mice in each group was measured. Finally, the area under curve (AUC) was calculated according to the blood glucose change at different measurement time, and the insulin sensitivity of mice in each group was measured according to a previous study [22].

### Western blotting

Total proteins were isolated from tissues or cells using RIPA lysis buffer (Beyotime, Shanghai, China). The protein concentration was measured using a BCA Protein Assay Kit (Beyotime). Then, 30 µg protein was seperated on 10% SDS-polyacrylamide gel and transferred to PVDF membrane (Merck Millipore). After that, the membranes were blocked using western blocking buffer (Beyotime). The primary antibodies (IRS-1, 1:1200; p-IRS-1, 1:1000; AKT, 1:1500; p-AKT, 1:800; AMPK, 1:1200; p-AMPK, 1:1000; UBE3A, 1:1500; incubated with primary antibodies overnight at 4 °C, followed by incubating with secondary antibody at 37 °C for 1 h. The bands were visualized using ECL detection kit (KeyGEN). GAPDH was the normalization.

For the determination of AMPK protein stability, the cells were treated with cycloheximide (CHX) to inhibit protein synthesis. The protein levels of AMPK in CHX treated cells for 0, 2, 4, 8 h were detected by western blot. According to the signal intensity of protein bands, the relative expression of each protein was calculated and the half-life curve of AMPK protein was drawn.

### Co-immunoprecipitation (Co-IP) assay

For Co-IP of endogenous proteins, the cells were washed twice with PBS (Sigma, USA), lysed with RIPA buffer (Beyotime). Then 500 µl cell lysates were incubated overnight at 4 ℃ with anti-UBE3A or anti-AMPK antibody. In order to obtain the immunocomplexes, the cell lysates were incubated 30 µl of protein-G agarose beads (Beyotime) at 4 ℃ for 4 h. Then after centrifugation, the supernatants were discarded to collect the agarose slurry. Next, the pellets were washed and resuspended in SDS gel-loading buffer. Finally, the bound proteins were analyzed by western blot analysis using anti-AMPK or anti-UBE3A.

For Co-IP of Exogenous proteins, lysates from HEK 293T cells treated with Flag-UBE3A or HA-AMPK. Then the immunocomplexes and anti-HA antibody or anti-Flag antibody were used for the Co-IP as above.

### Statistical analysis

The data were in this study was shown as mean ± SD. The statistical analysis was performed using SPSS 22.0 (SPSS, USA) and the figures were generated using PRISM 7.0 (GraphPad). Data were tested for normality of distribution and were confirmed to meet normality. Then, the difference between two groups was analyzed using Student’s t-test, and the difference between multiple groups was analyzed using one-way analysis of variance (ANOVA). Statistical significance was considered to be P < 0.05.

## Results

### UBE3A was up-regulated in PCOS progression

**Fig. 1:**
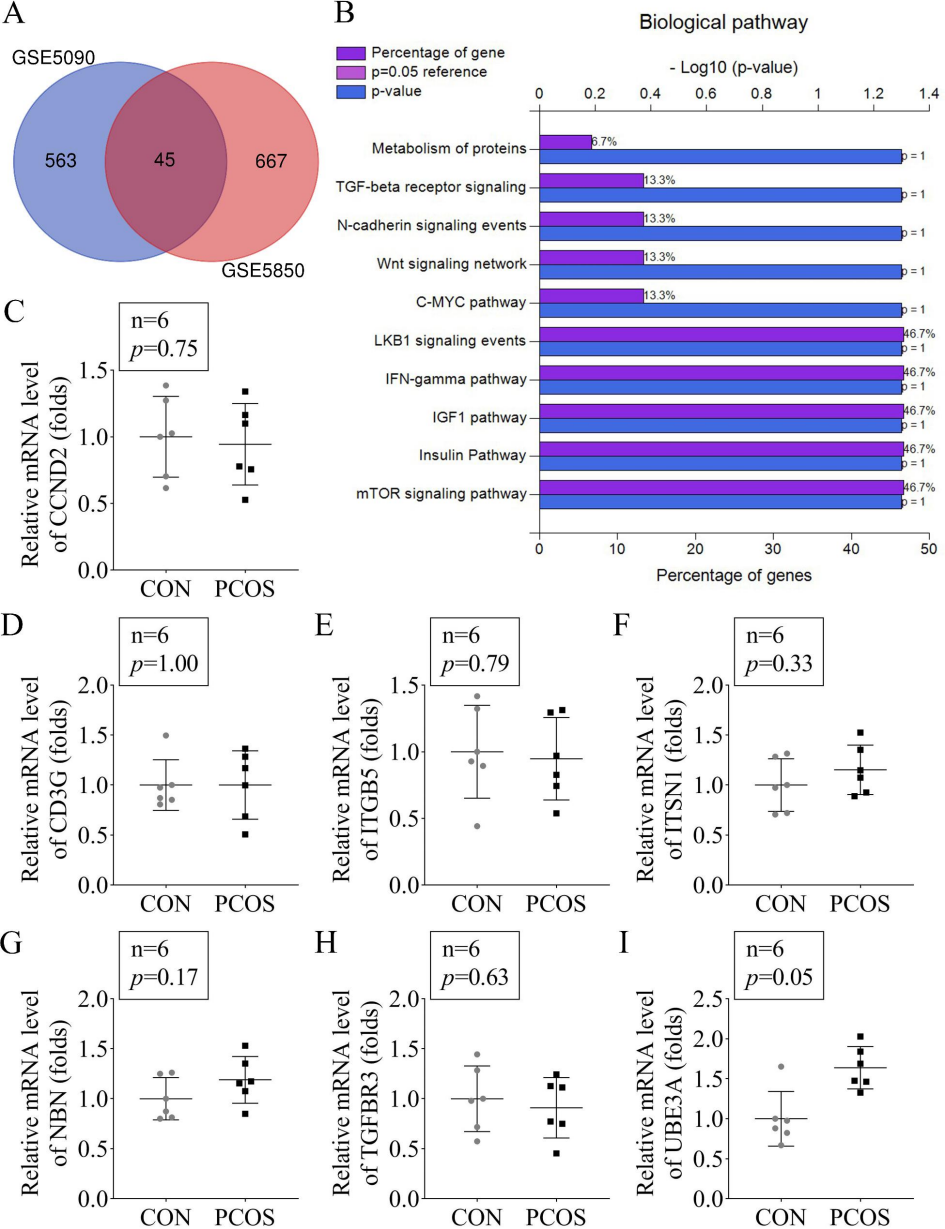
UBE3A was up-regulated in PCOS rats **(A)** The up-regulated genes in the PCOS patients obtained from the GSE5090 and GSE5850 data set. **(B)** The KEGG analysis of the 45 co-differentially up-regulatede genes. (C-I) The mRNA levels of genes enriched in the LKB1 signaling in the serum of the PCOS rats were detected by RT-qPCR.

Through the GSE5090 and GSE5850 data set, we obtained 45 co-significantly up-regulated genes in PCOS (Fig. 1A). Then, through the KEGG pathway analysis, the 45 up-regulated genes enriched signaling pathways were showed in Fig. 1B. Then we analyzed the mRNA levels of genes enriched in the LKB1 signaling in the serum of the PCOS rat (Fig. 1C-I). The PCR results showed that just UBE3A was significantly increased in the PCOS rat (*p* = 0.05). Therefore, UBE3A was selected for the next experiments after being the only molecule found significantly overexpressed after PCR.

### IR up-regulated the UBE3A levels in PCOS rats

**Fig. 2:**
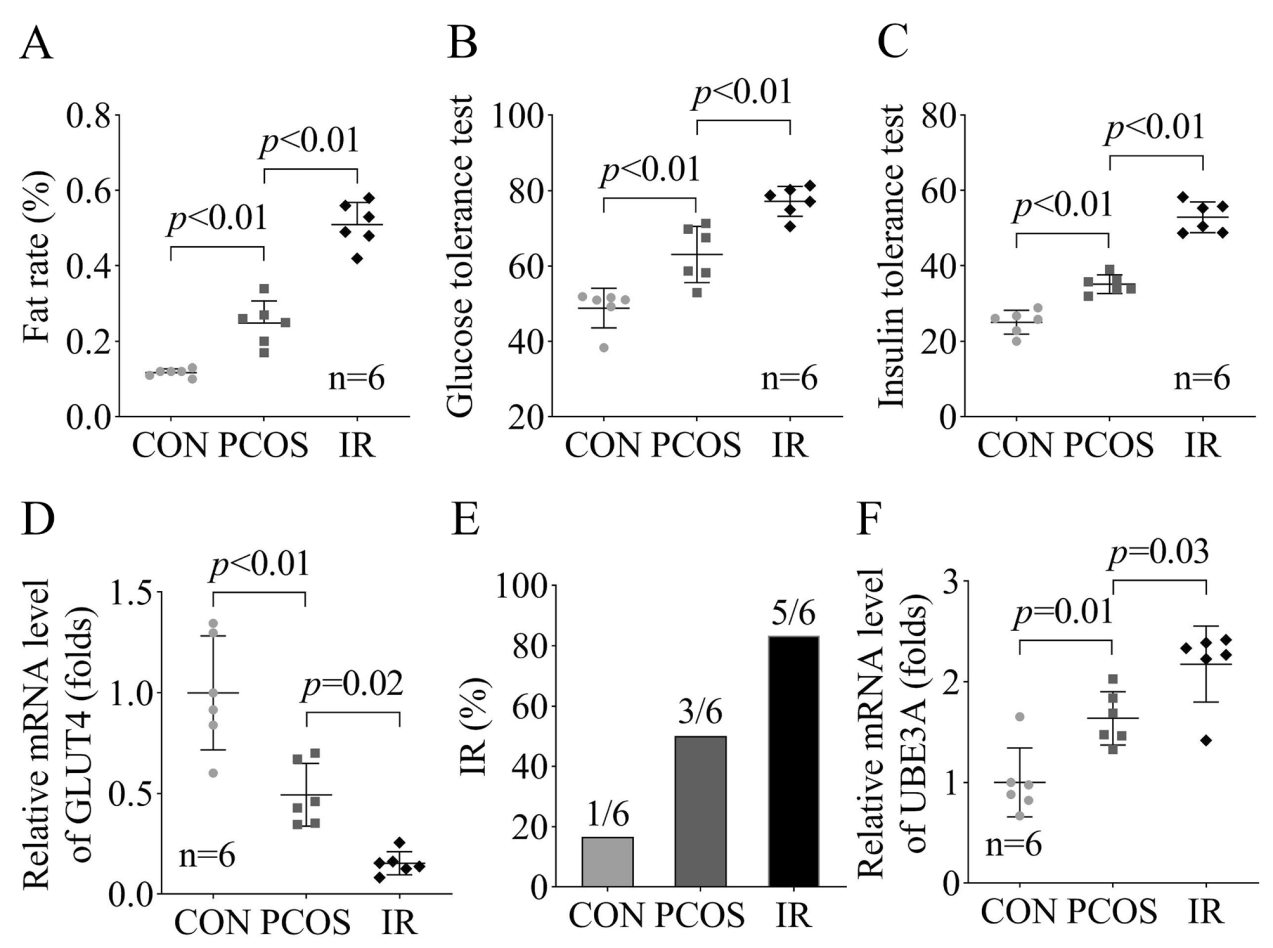
IR up-regulated the UBE3A levels in PCOS rats The fat rate **(A)**, glucose tolerance **(B)** and insulin tolerance **(C)** in the PCOS and PCOS + IR rats. **(D)** The GLUT4 mRNA levels were in the PCOS and PCOS + IR rats were detected by RT-qPCR. **(E)** The number of IR rats in each group were calculated. **(F)** The UBE3A mRNA levels were in the PCOS and PCOS + IR rats were detected by RT-qPCR.

Previous study has found that IR plays an important role in the occurrence and development of PCOS, leading to the continuous aggravation of the PCOS patient’s condition. Therefore, we establish the PCOS and IR rat models through the DHT injection and HFD treatment to explore relationship between IR and PCOS [5]. We found that the fat rate (Fig. 2A), glucose tolerance (Fig. 2B) and insulin tolerance (Fig. 2C) were significantly increased in the PCOS rats (*p* < 0.01), which was further aggravated in the PCOS + IR rats (*p* < 0.01). Besides, we found that GLUT4 levels were significantly decreased in PCOS rats (*p* < 0.01), and also aggravated in the PCOS + IR rats (*p* = 0.02, Fig. 2D). Furthermore, we found that there is one rat developed IR in the control group, 3 rats developed IR in the PCOS group, and 5 rats developed IR in the PCOS + IR group (Fig. 2E). Besides, the UBE3A mRNA levels were significantly increased the PCOS rats (*p =* 0.01), and further increased in the PCOS + IR rats (*p* = 0.03, Fig. 2F). These results indicated that IR significantly increased the UBE3A levels, which further promoted the PCOS progression.

### UBE3A knockdown relieved the IR progression in the PCOS rats

**Fig. 3:**
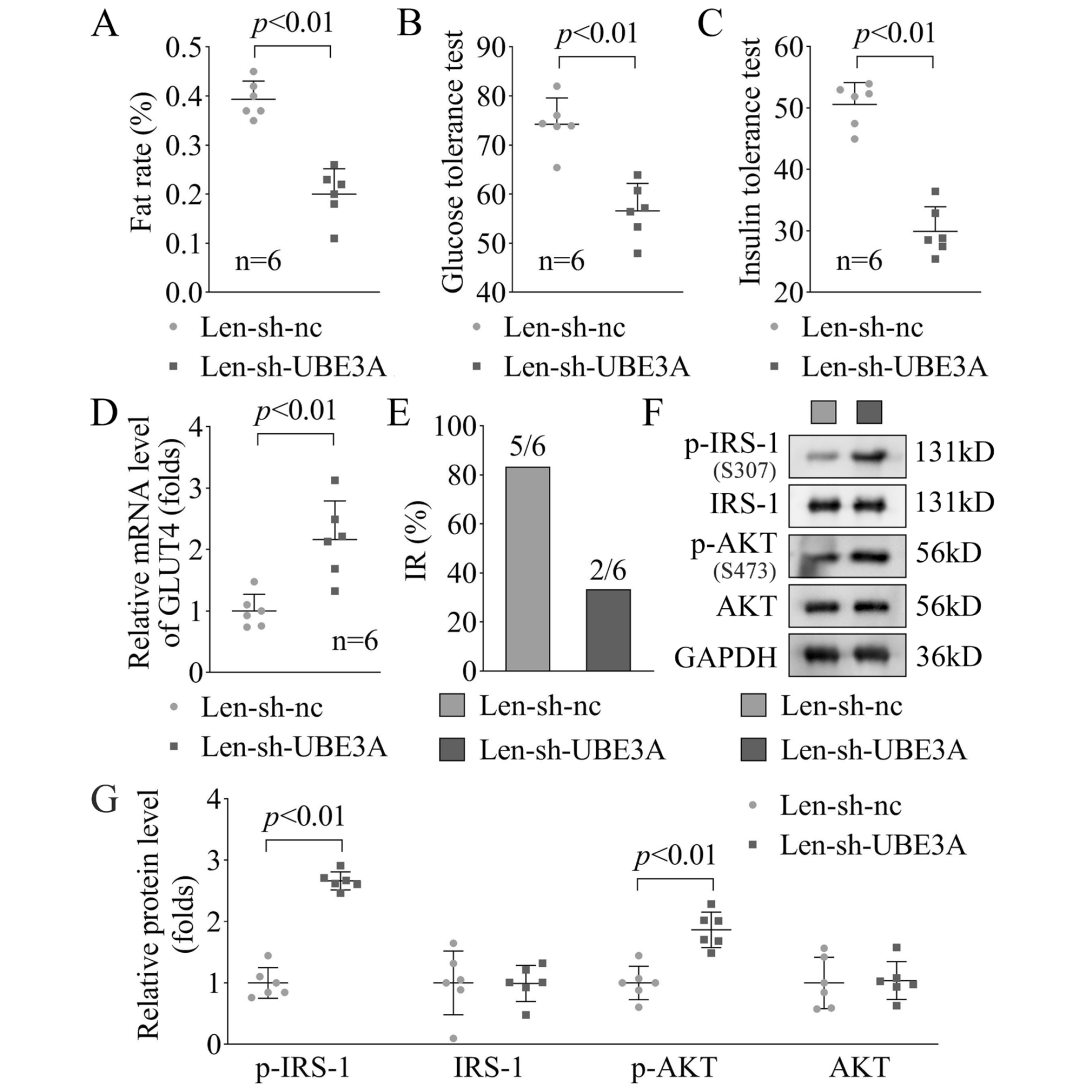
UBE3A knockdown relieved the IR progression in the PCOS rats After UBE3A knockdown, the fat rate **(A)**, glucose tolerance **(B)** and insulin tolerance **(C)** in the PCOS + IR rats. **(D)** The GLUT4 mRNA levels were in the PCOS + IR rats were detected by RT-qPCR. **(E)** The number of IR rats in each group were calculated. **(F & G)** The p-IRS1 and p-AKT protein levels were in the PCOS + IR rats were detected by western blot

Subsequently, we further explore the role of UBE3A knockdown in the PCOS + IR rats. After UBE3A knockdown, we found that fat rate (*p* < 0.01, Fig. 3A), glucose tolerance (*p* < 0.01, Fig. 3B) and insulin tolerance (*p* < 0.01, Fig. 3C) were significantly decreased in the PCOS + IR rats. Additionally, the GLUT4 levels were significantly increased in PCOS + IR rats after UBE3A knockdown (*p* < 0.01, Fig. 3D). Then we found that after UBE3A knockdown, the IR rats were decreased (n = 2) compared with the Len-sh-nc group (n = 5) (Fig. 3E). What’s more, the p-IRS1 and p-AKT levels in the PCOS + IR rats were significantly up-regulated after UBE3A knockdown (Fig. 3F-G). These results suggested that UEB3A knockdown effectively inhibited the IR in PCOS progression.

### UBE3A knockdown increased the phosphorylation of AMPK through decreasing the ubiquitination of AMPK

**Fig. 4:**
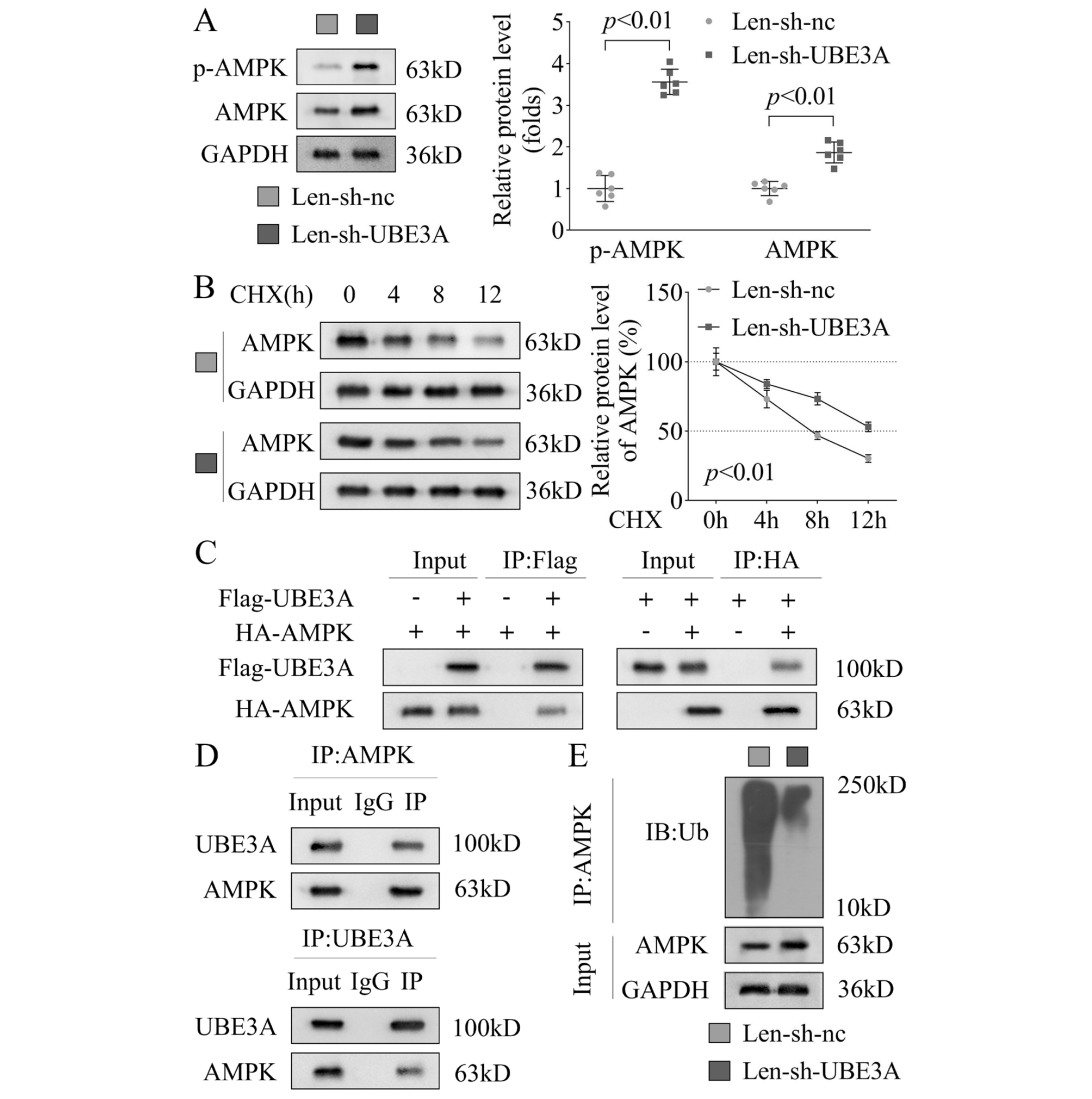
UBE3A knockdown increased the phosphorylation of AMPK through decreasing the ubiquitination of AMPK in the KGN cells **(A)** The protein levels of p-AMPK and AMPK were detected by western blot after UBE3A knockdown. **(B)** The protein stability of AMPK detected by western blot after UBE3A knockdown. **(C)** Exogenous interaction between AMPK and UBE3A was determined using co-IP with anti-Flag or anti-HA antibodies co-transfected with Flag-UBE3A and HA-AMPK. **(D)** Endogenous interaction between AMPK and UBE3A was determined using co-IP with anti-AMPK or anti-UBE3A antibodies. **(E)** The ubiquitination of AMPK after UBE3A knockdown was detected by western blot

LKB1 signaling has been demonstrated to be closely related to AMPK [23]. Then we found that after UBE3A knockdown, the phosphorylation of AMPK, total AMPK protein levels (Fig. 4A) and protein stability (Fig. 4B) were significantly increased in the KGN cells. Then we further analyzed the relationship between AMPK and UBE3A. Through the co-IP assay, we verified the interaction between UBE3A and AMPK at both the exogenous (Fig. 4C, in the KGN cells) and endogenous (Fig. 4D, in the HEK 293T cells) protein levels. Furthermore, we found that UBE3A knockdown decreased the ubiquitination of AMPK and increased total AMPK protein levels (Fig. 4E). These results indicated that UBE3A knockdown might activate the LKB1 signaling pathway through decreasing the ubiquitination of AMPK.

### AMPK knockdown reversed the role of UBE3A knockdown in the PCOS rats

**Fig. 5:**
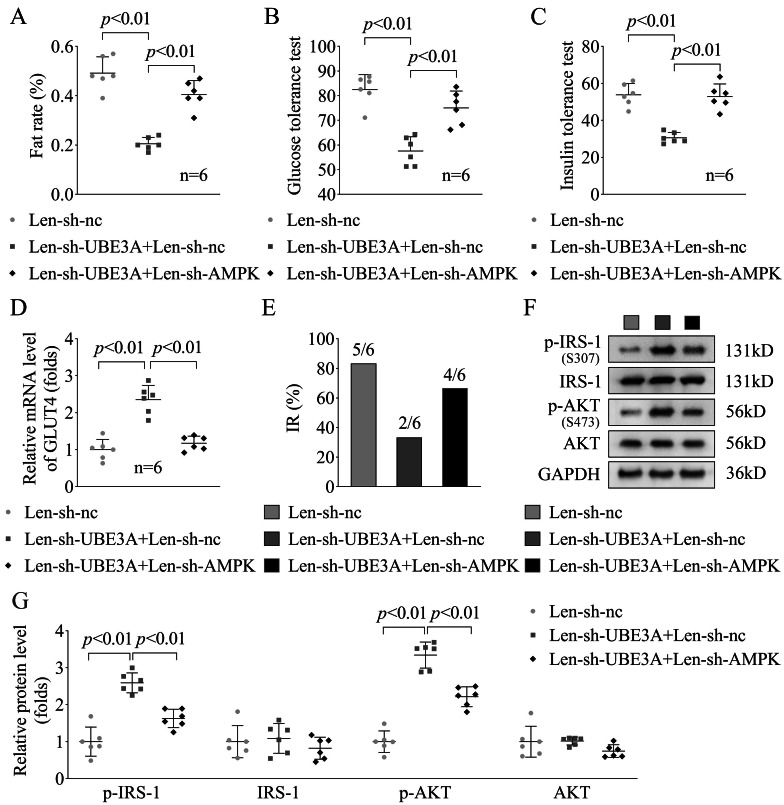
AMPK knockdown reversed the role of UBE3A knockdown in the PCOS rats After UBE3A and AMPK knockdown, the fat rate **(A)**, glucose tolerance **(B)** and insulin tolerance **(C)** in the PCOS + IR rats. **(D)** The GLUT4 mRNA levels were in the PCOS + IR rats were detected by RT-qPCR. **(E)** The number of IR rats in each group were calculated. **(F & G)** The p-IRS1 and p-AKT protein levels were in the PCOS + IR rats were detected by western blot

Finally, we found that after AMPK knockdown, that fat rate (*p* < 0.01, Fig. 5A), glucose tolerance (*p* < 0.01, Fig. 5B) and insulin tolerance (*p* < 0.01, Fig. 5C) were significantly increased in the UBE3A knockdown PCOS + IR rats. Additionally, the GLUT4 levels were significantly decreased in UBE3A knockdown PCOS + IR rats after AMPK knockdown (*p* < 0.01, Fig. 5D). Then we found that after AMPK knockdown, the IR rats were increased (n = 4) compared with the Len-sh-UBE3A + Len-sh-nc group (n = 2) (Fig. 5E). What’s more, the p-IRS1 and p-AKT levels in the UBE3A knockdown PCOS + IR rats were significantly down-regulated after AMPK knockdown (Fig. 5F-G). These results indicated that UBE3A knockdown inhibited the PCOS progression through increasing the AMPK levels.

## Discussion

In this study, we found that UBE3A regulated the IR development in the PCOS rats through targeting AMPK expression levels. Mechanistically, UBE3A knockout enhanced phosphorylation of AMPK through decreasing the ubiquitination of AMPK. AMPK knockdown reversed the role of UBE3A knockdown in the PCOS + IR rats.

PCOS is one of the important risk factors of ovulatory dysfunction infertility in women, the pathogenesis of which is relatively complex. At present, many researchers believe that high androgen is the key cause of PCOS, and high level of androgen can stimulate the decomposition of visceral fat and promote the islet of pancreas β Cells release insulin and induce IR in the body [7, 24, 25]. IR promotes the rapid internalization of activated insulin receptor into cytosol, which blocked the insulin signaling and aggravated the disease development [26, 27]. Previous studies also found that the expression of glucose transporter 4 (GLUT4) and post-insulin receptor signal transduction molecules in the endometrium of PCOS patients with IR were decreased [28–30]. Uche et al. [31] found that PCOS women demonstrated increased IR in adipose tissue, which is closely associated with whole-body IR. Liu et al. [32] demonstrated that modified Cangfu Daotan decoction may play a role in improving ovarian function in PCOS-IR rats by downregulating upregulating the gene expression of IRS-1/GLUT4 in the insulin signaling pathway in the inflammatory environment. Similarly, here, we found that GLUT4 were significantly decreased in the PCOS rats (*p* < 0.01), and IR was also happened. After HFD treatment, the GLUT4 levels were further decreased (*p* = 0.02) and IR was aggravated. Therefore, we speculated that paying attention to IR may be the key to the treatment of PCOS.

UBE3A, a ubiquitin modified gene, has been demonstrated to accelerate the target gene degradation through enhancing the ubiquitination in many diseases. For example, Wang et al. [17] found that PTPA, an activator of protein phosphatase 2 A, was a bona fide ubiquitin ligase substrate of UBE3A. Low levels of UBE3A decreased the ubiquitination of PTPA, which further accelerated PP2A holoenzyme assembly, and enhanced its activity in angelman syndrome. Additionally, in esophageal cancer, Zheng et al. [33] demonstrated that UBE3A was increased and promoted the malignant behavior of esophageal cancer cells through degrading ZNF185. However, the effects of UBE3A on IR of PCOS remains unclear. In this study, through the bioinformatic analysis, we found that UBE3A was significantly up-regulated in PCOS, which was further confirmed in PCOS patients (*p* = 0.05) and rats (*p* = 0.01). Additionally, UEB3A silencing relieved the IR in PCOS rats. These results preliminarily revealed the role of UEB3A in PCOS. Interestingly, through the KEGG pathway analysis, UEB3A was demonstrated to be enriched in LKB1 signaling. LKB1, as the upstream kinase of AMPK, can directly phosphorylate AMPK to participate in glycolipid metabolism and regulate the occurrence and development of IR [23]. Recent researches have demonstrated that AMPK participated in the PCOS progression. Heidy et al. [34] suggested that myo-inositol inhibited the IR in PCOS through increasing the AMPK levels. Selenay et al. [35] demonstrated that metformin and resveratrol treatment played the antioxidant and antiinflammatory roles in PCOS patients through enhancing the AMPK levels. Combined with our bioinformatic analysis, we speculated whether UEB3A participated in the occurrence and development of IR in PCOS by regulating the expression of AMPK. Here, we found that UEB3A knockdown decreased the ubiquitination levels of AMPK, while the phosphorylation of AMPK was enhanced. This implied that the degradation role of UEB3A in AMPK is achieved by inhibiting its phosphorylation. Importantly, through the exogenous and endogenous Co-IP assay, the interaction between UEB3A and AMPK was confirmed. The rescue experiments demonstrated that AMPK silencing reversed the effects of UEB3A on the IR in the PCOS rats.

In conclusion, our study indicated that UBE3A expression is elevated in PCOS progression. UBE3A expression is closely related to IR. UBE3A knockdown inhibited the IR in PCOS rats through enhancing the AMPK levels by ubiquitination. Our study indicated that UBE3A might become a potential biological target for the clinical treatment of PCOS in future.

## Electronic supplementary material

Below is the link to the electronic supplementary material.

Supplementary Material 1

## Data Availability

The datasets used and/or analyzed during the current study are available from the corresponding author on reasonable request.
