# Supplementary material for: Effects of UBE3A on the insulin resistance in polycystic ovary syndrome through the ubiquitination of AMPK
Source: BMC Endocr Disord. 2023 Jul 17;23:152. doi: 10.1186/s12902-023-01400-8 (PMC10351129; doi:10.1186/s12902-023-01400-8)
Supplement: Supplementary file 1 — Supplementary Material 1 [file 12902_2023_1400_MOESM1_ESM.docx]

| **Figure3** | |
| --- | --- |
| **p-IRS-1** | **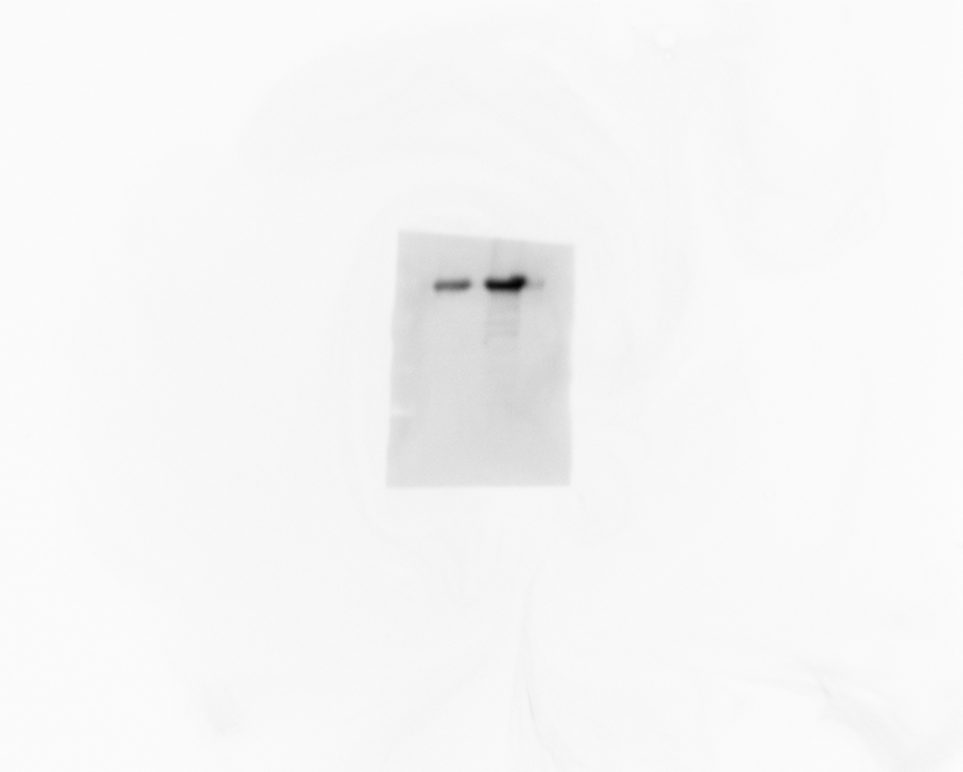** |
| **IRS-1** | **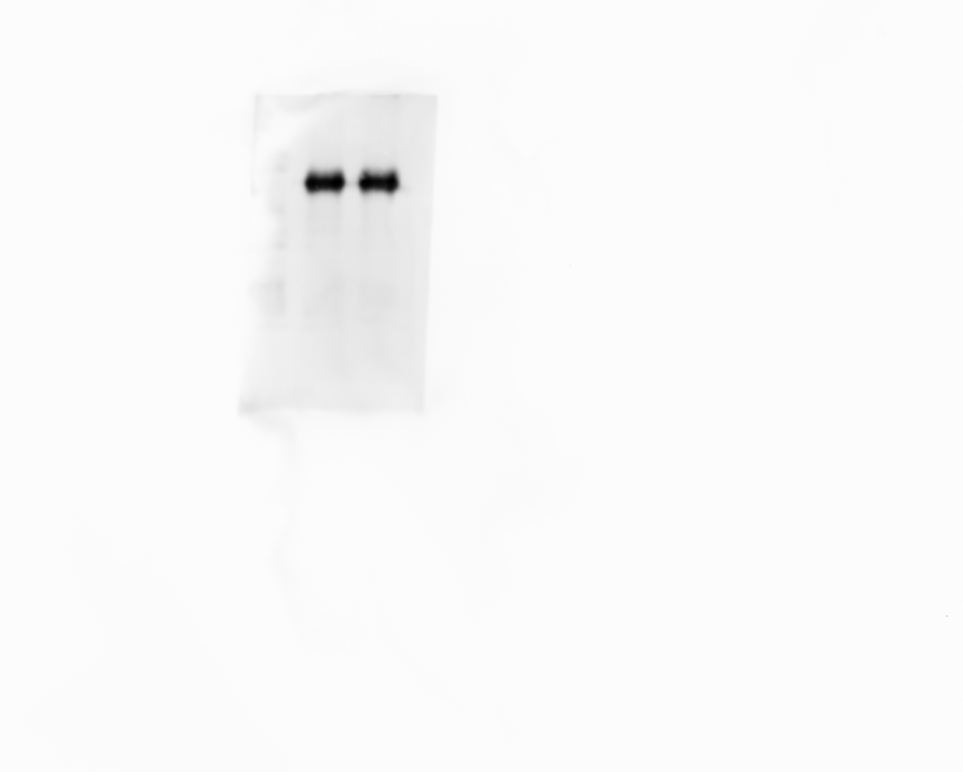** |
| **p-AKT** | **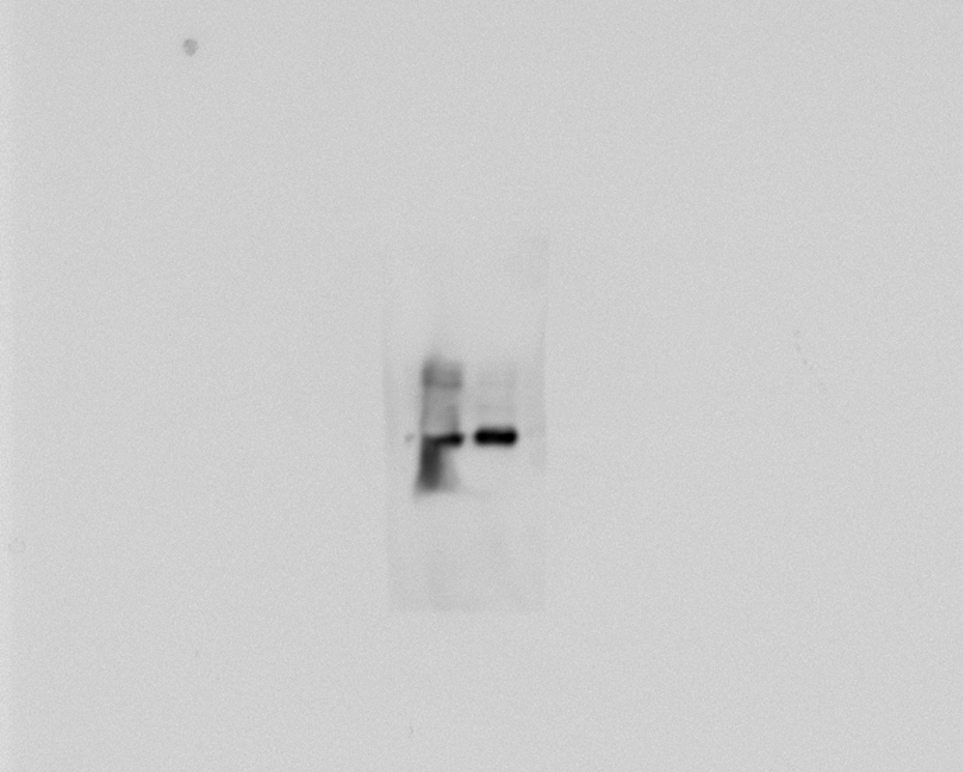** |
| **AKT** | **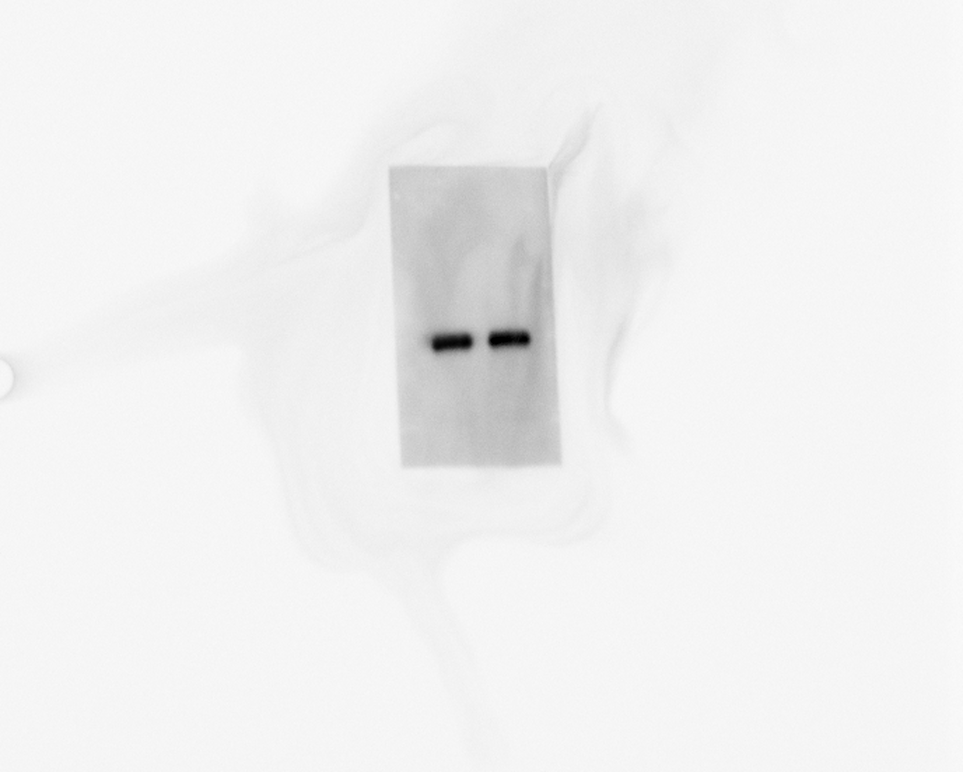** |
| **GAPDH** | **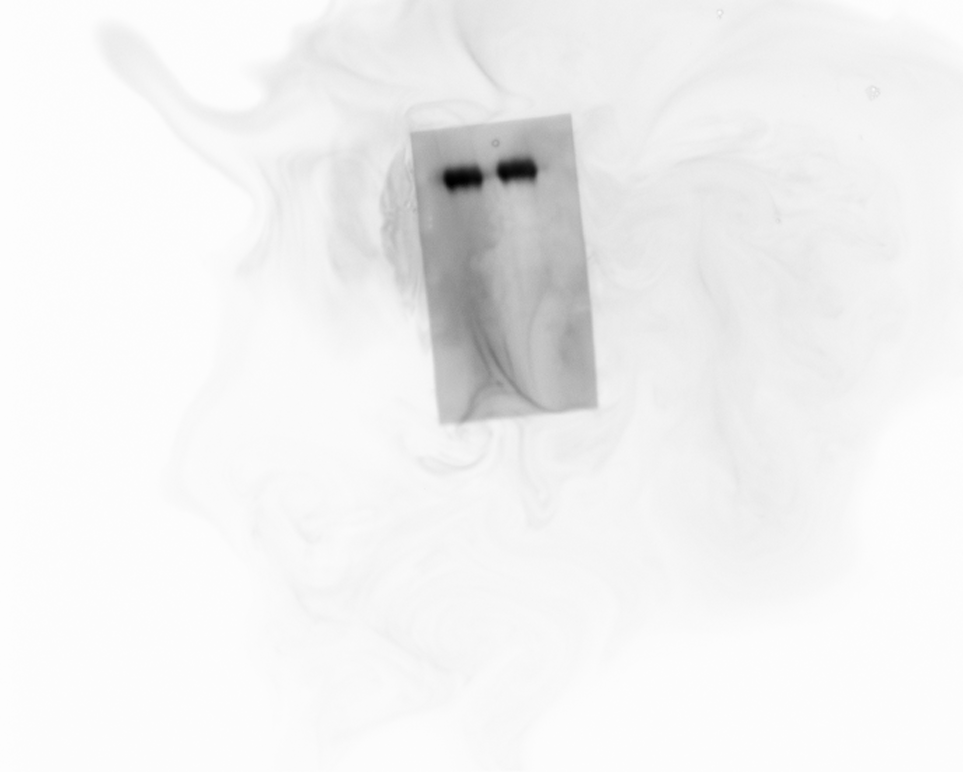** |

| **Figure4-A** | |
| --- | --- |
| **p-AMPK** | **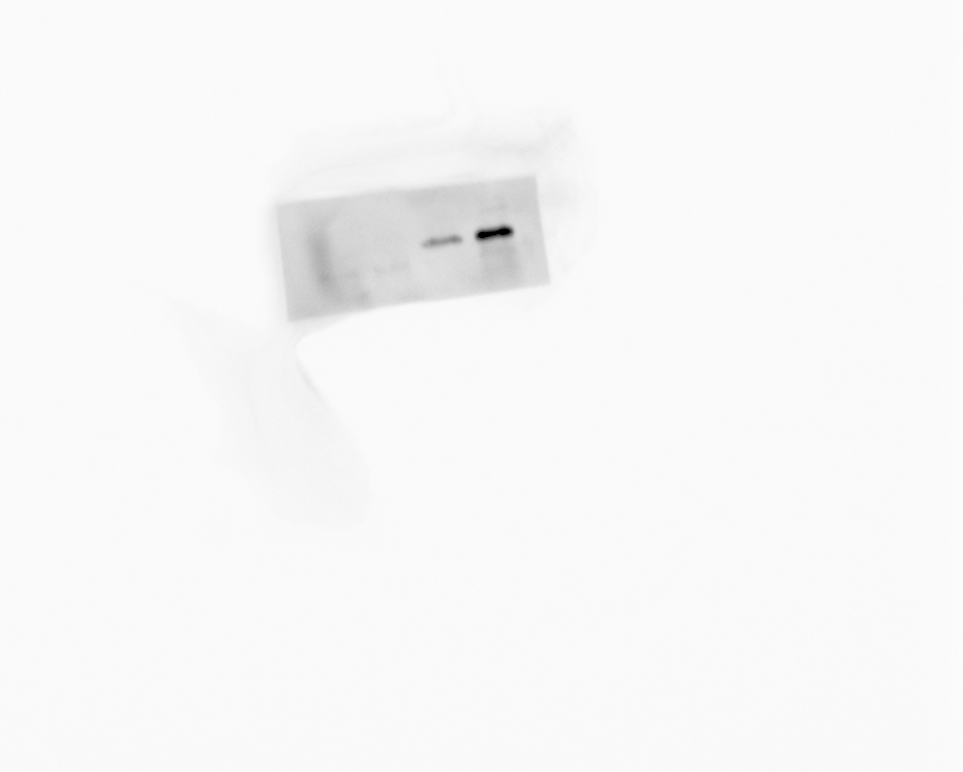** |
| **AMPK** | **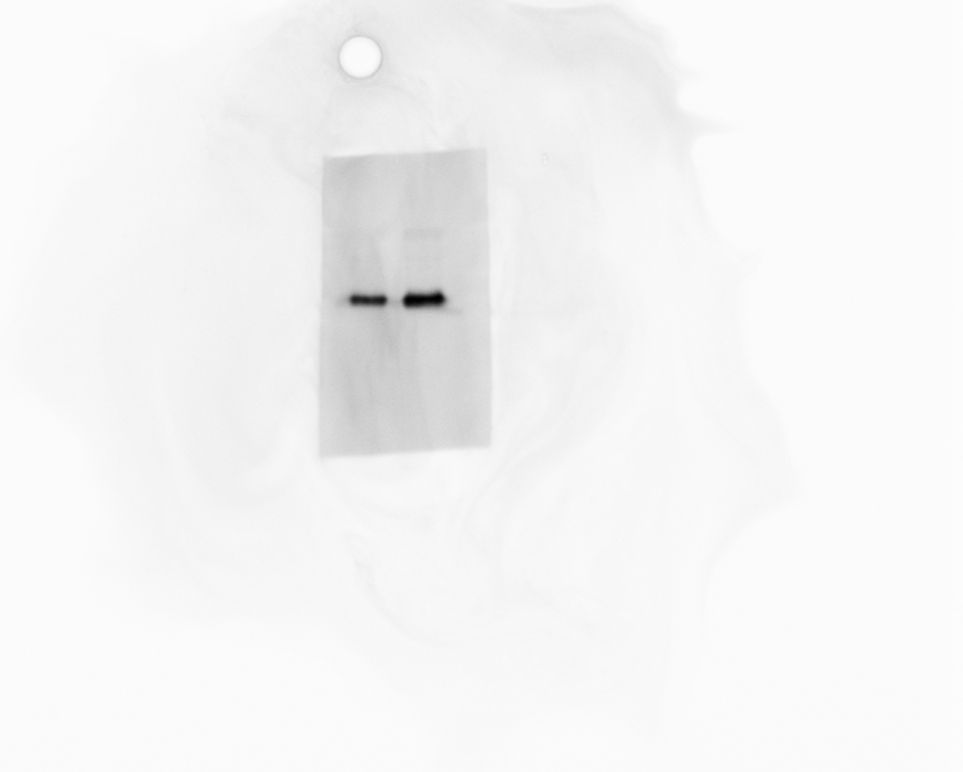** |
| **GAPDH** | **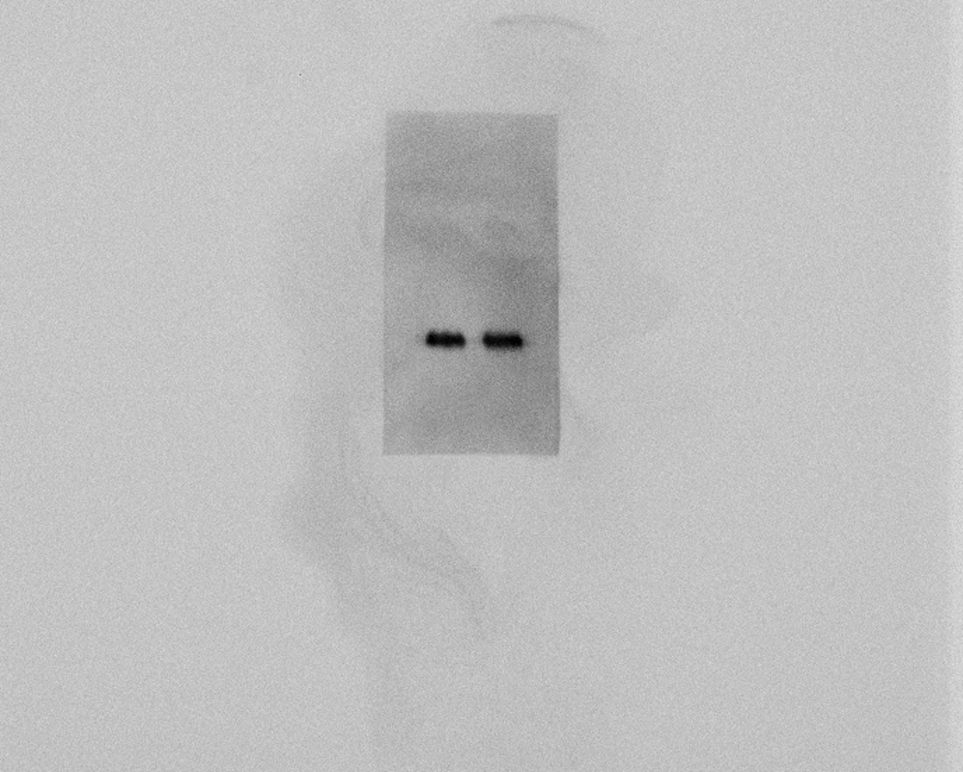** |

| **Figure4-B** | |
| --- | --- |
| **AMPK** | **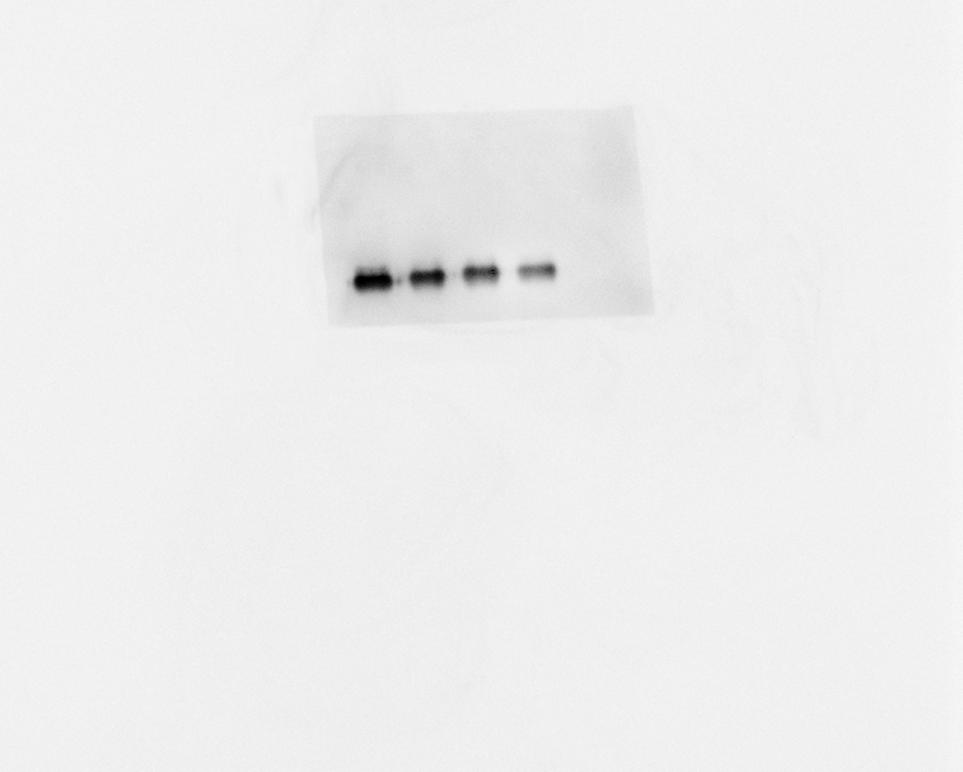** |
| **GAPDH** | **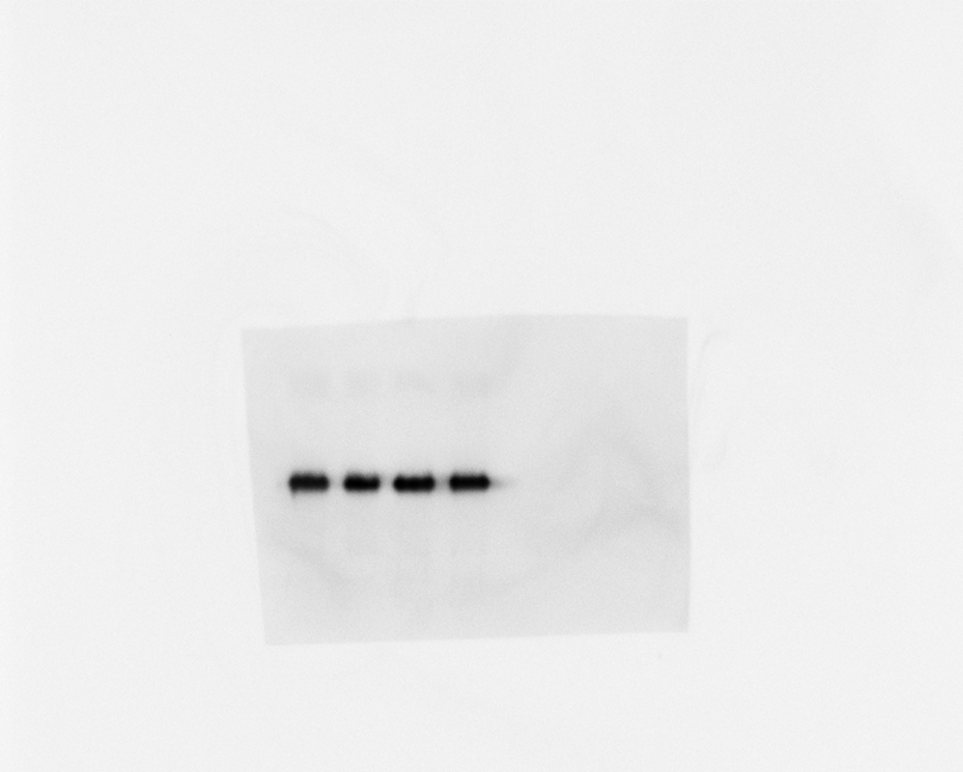** |
| **AMPK** | **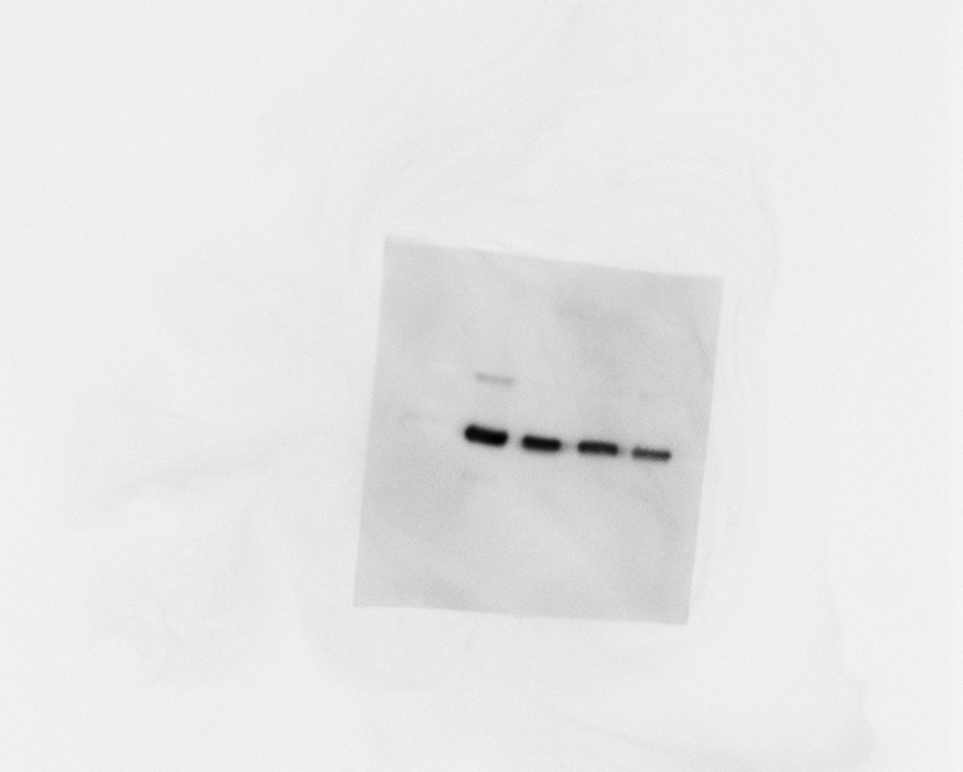** |
| **GAPDH** | **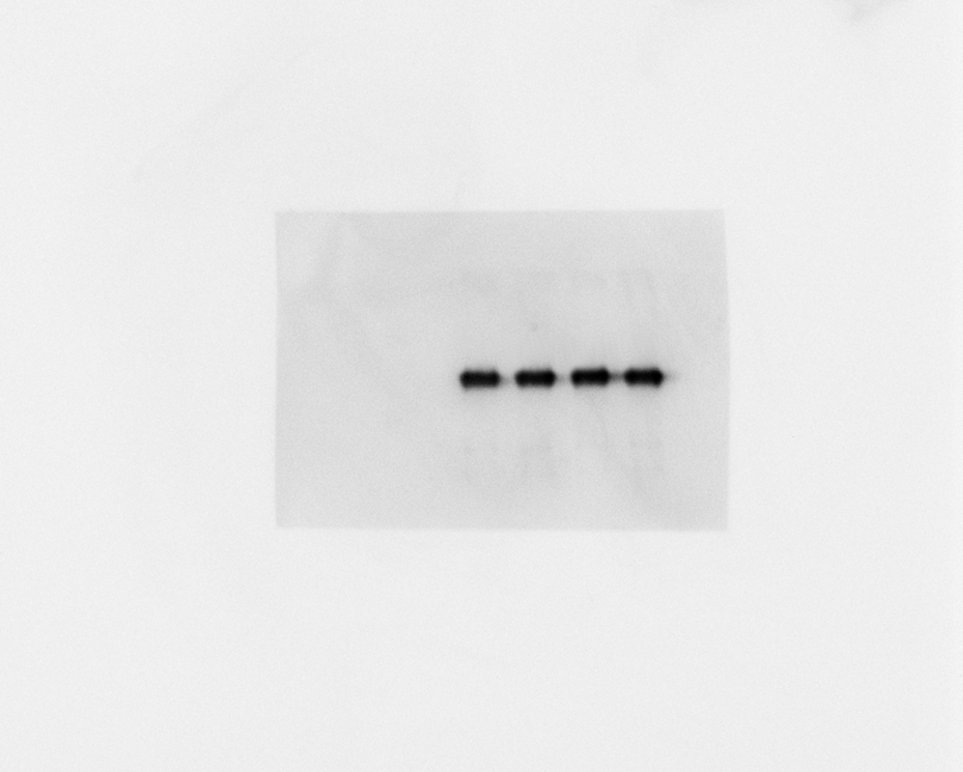** |

| **Figure4-C** | |
| --- | --- |
| **Flag-UBE3A**  **IP:Flag** | **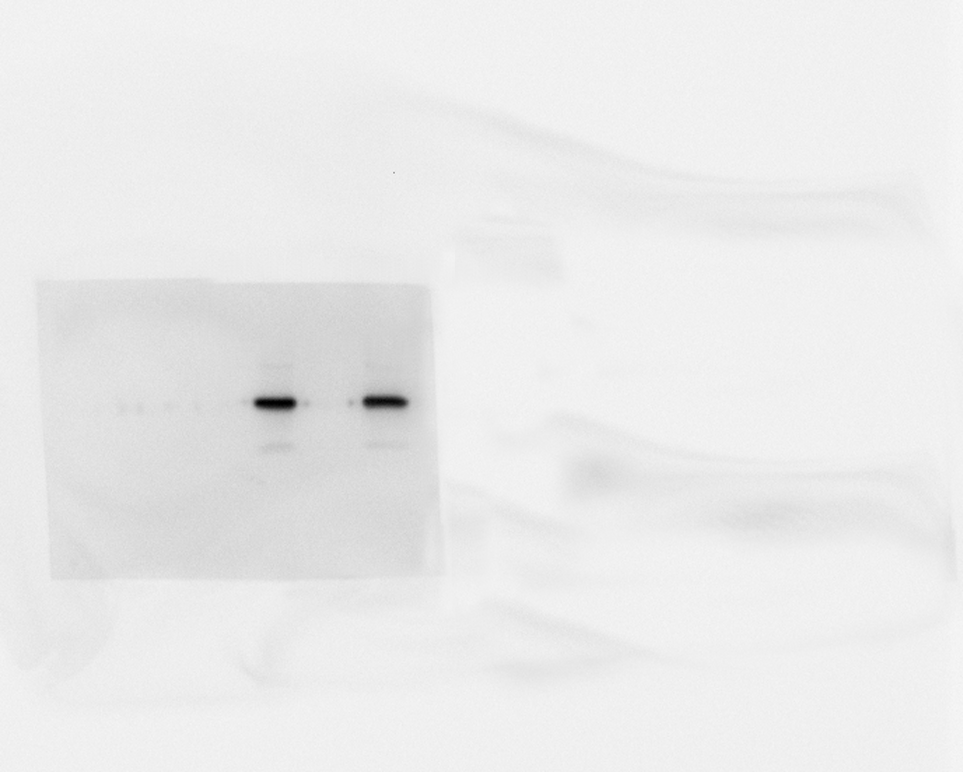** |
| **HA-AMPK**  **IP:Flag** | **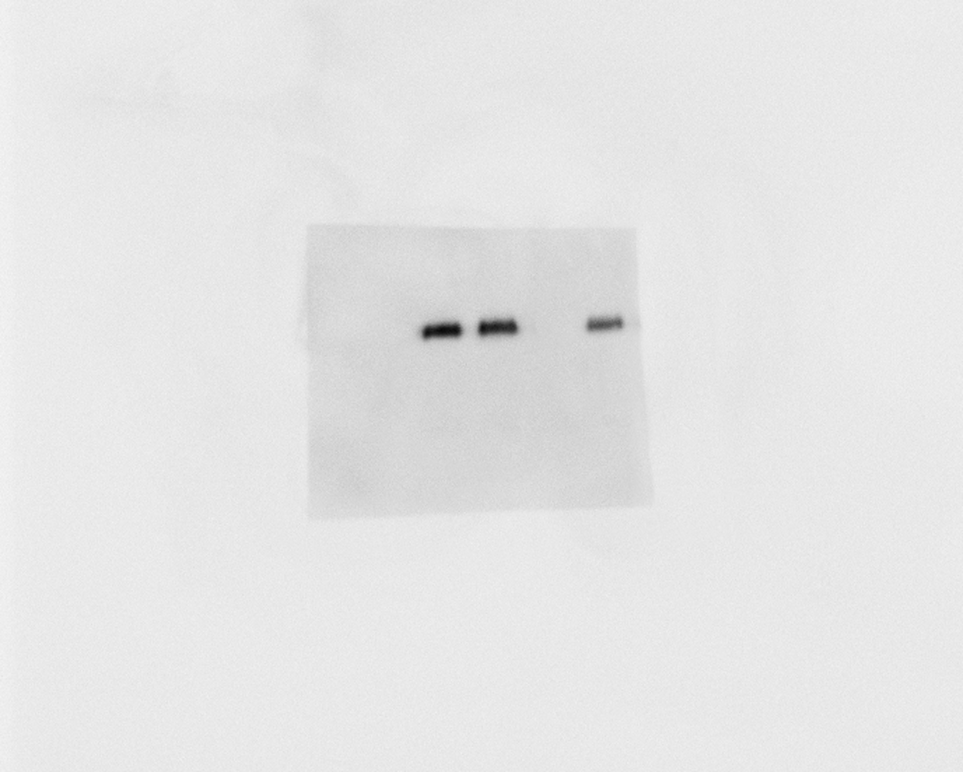** |
| **Flag-UBE3A**  **IP:HA** | **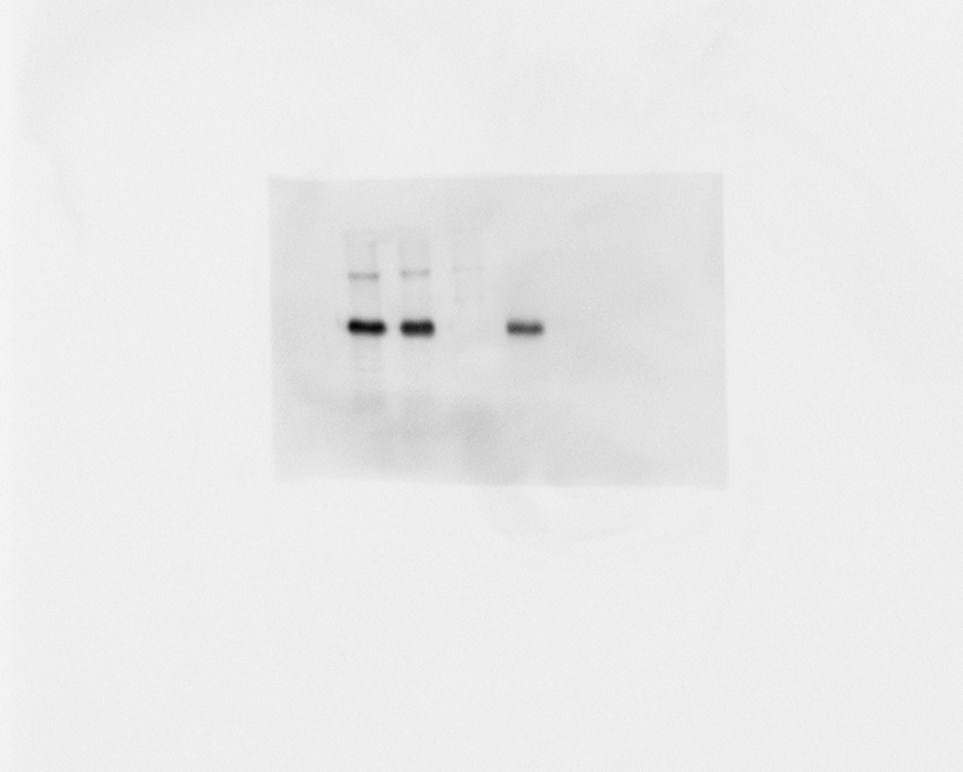** |
| **HA-AMPK**  **IP:HA** | **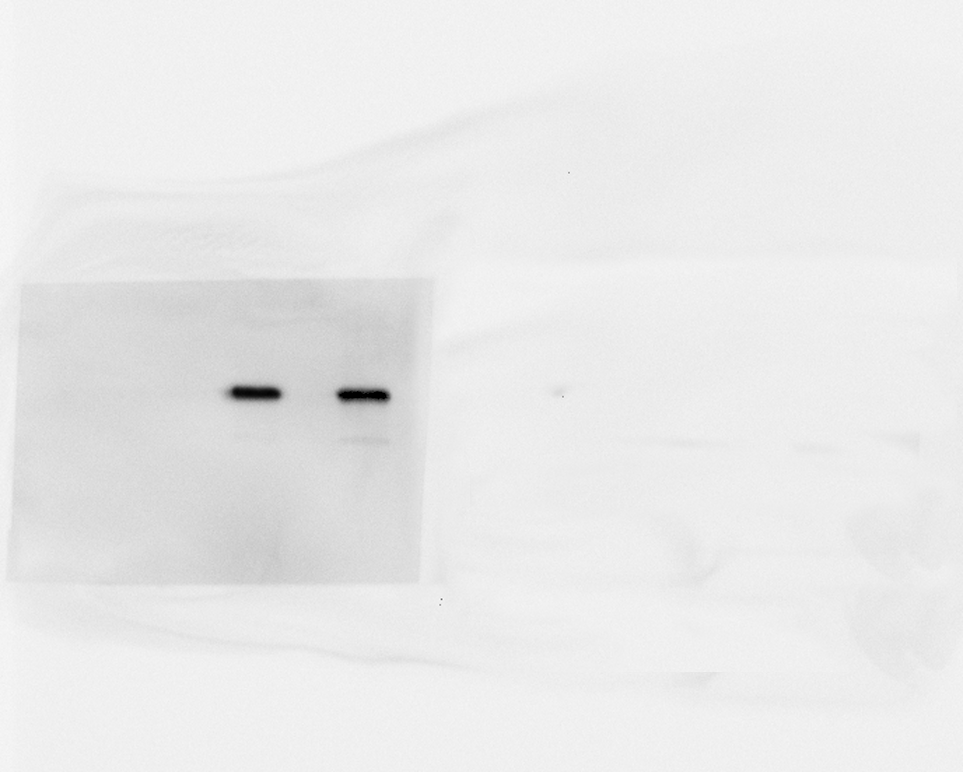** |

| **Figure4-D** | |
| --- | --- |
| **UBE3A**  **IP:AMPK** | **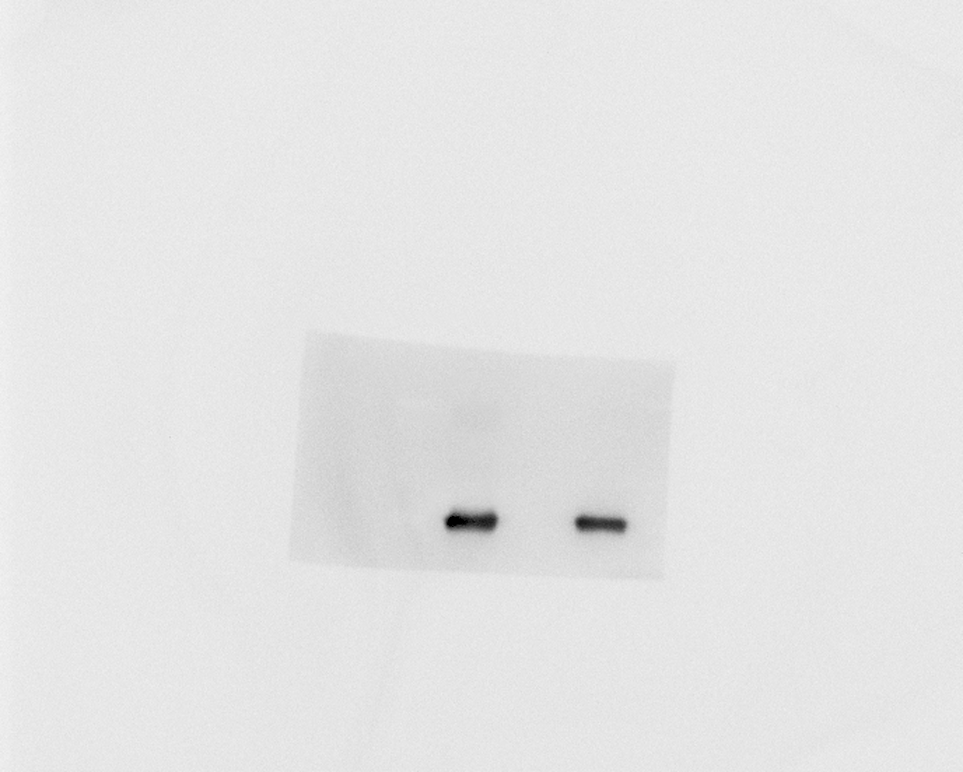** |
| **AMPK**  **IP:AMPK** | **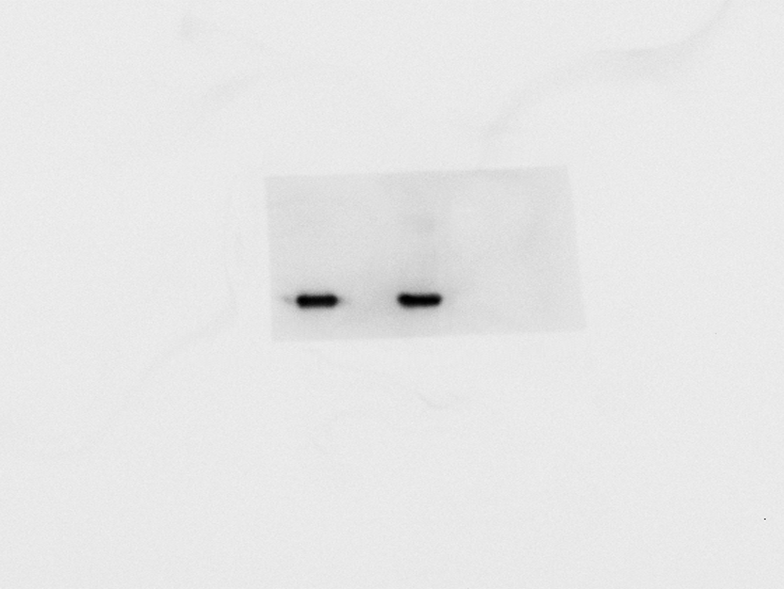** |
| **UBE3A**  **IP:UBE3A** | **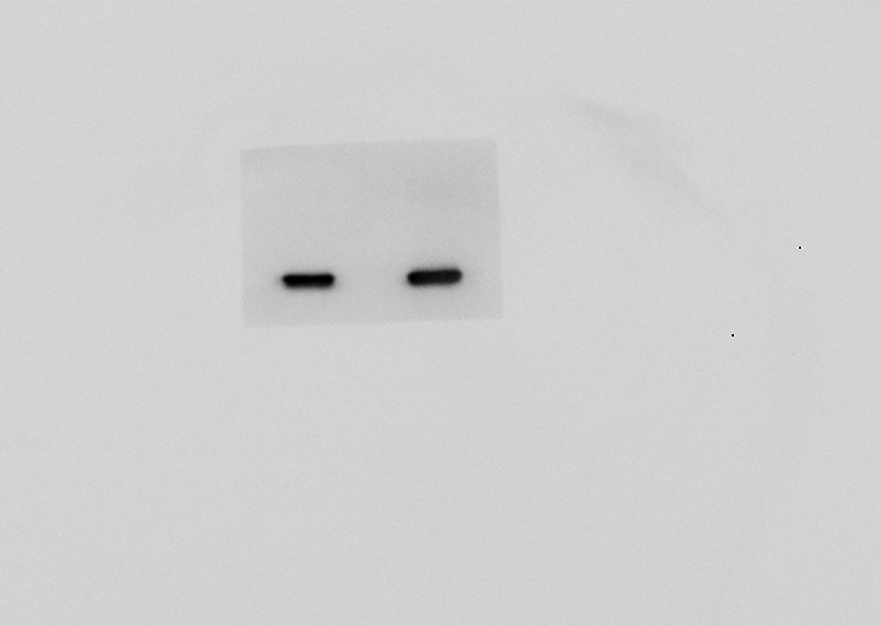** |
| **AMPK**  **IP:UBE3A** | **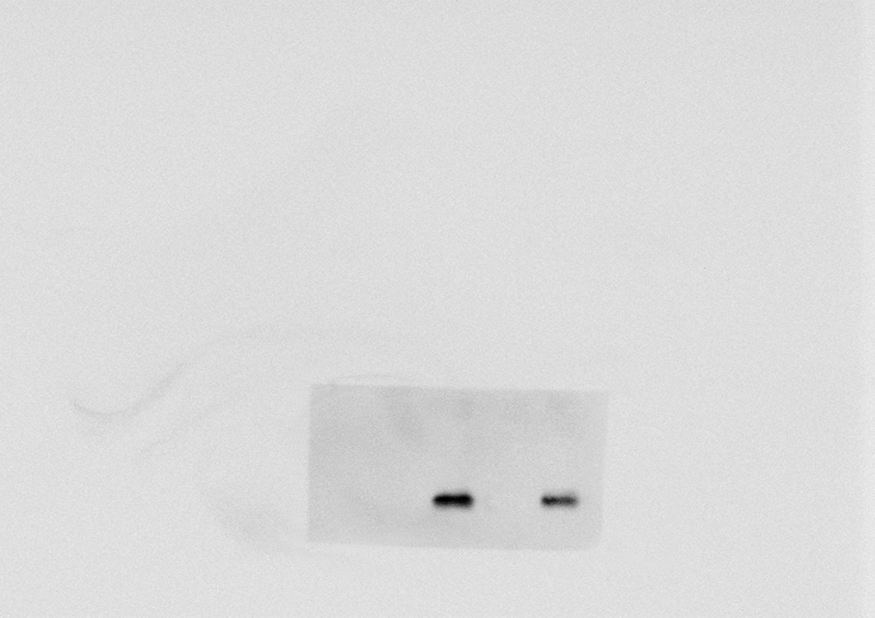** |

| **Figure4-E** | |
| --- | --- |
| **AMPK** | **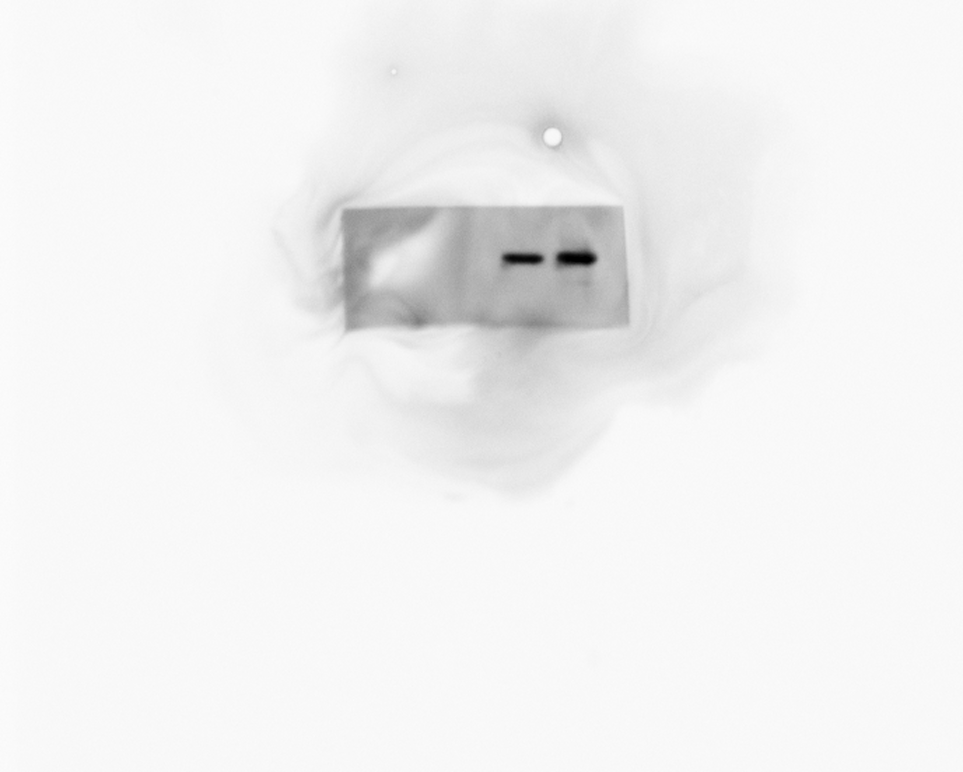** |
| **GAPDH** | **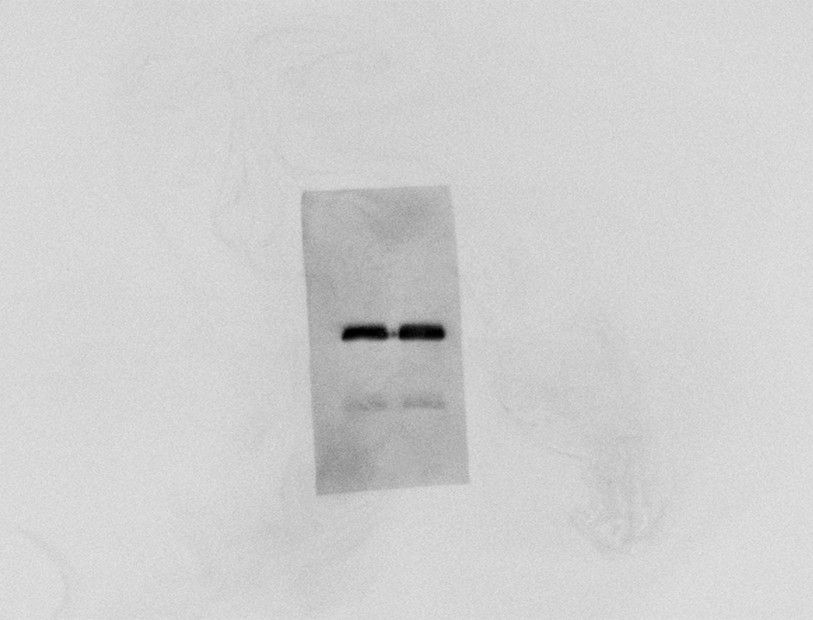** |

| **Figure5** | |
| --- | --- |
| **p-IRS-1** | **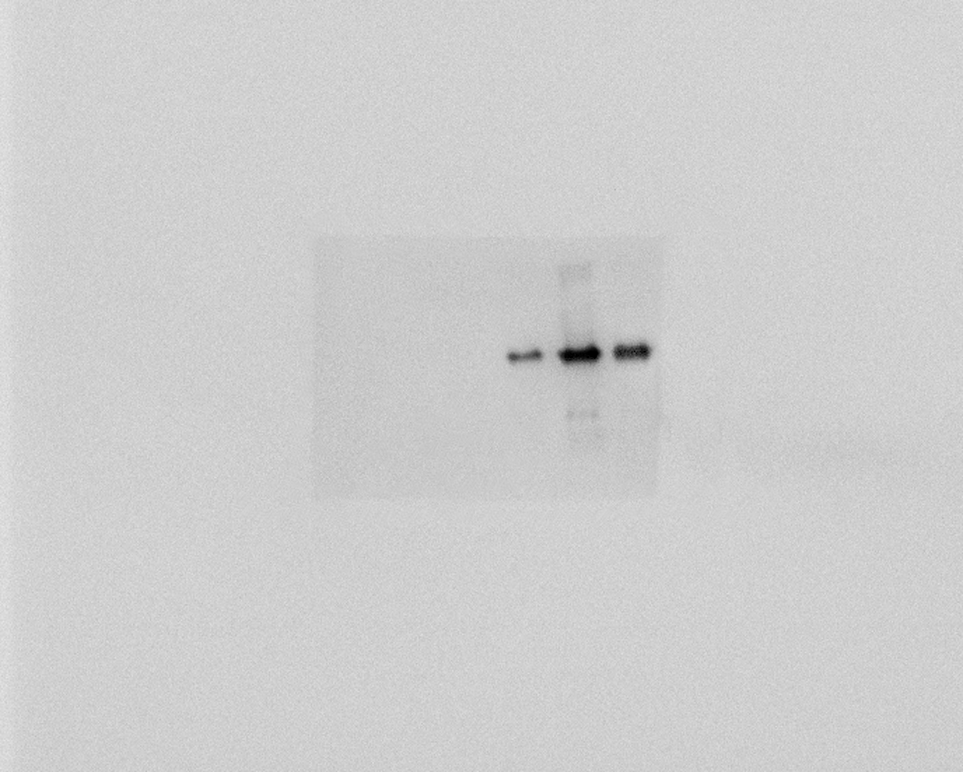** |
| **IRS-1** | **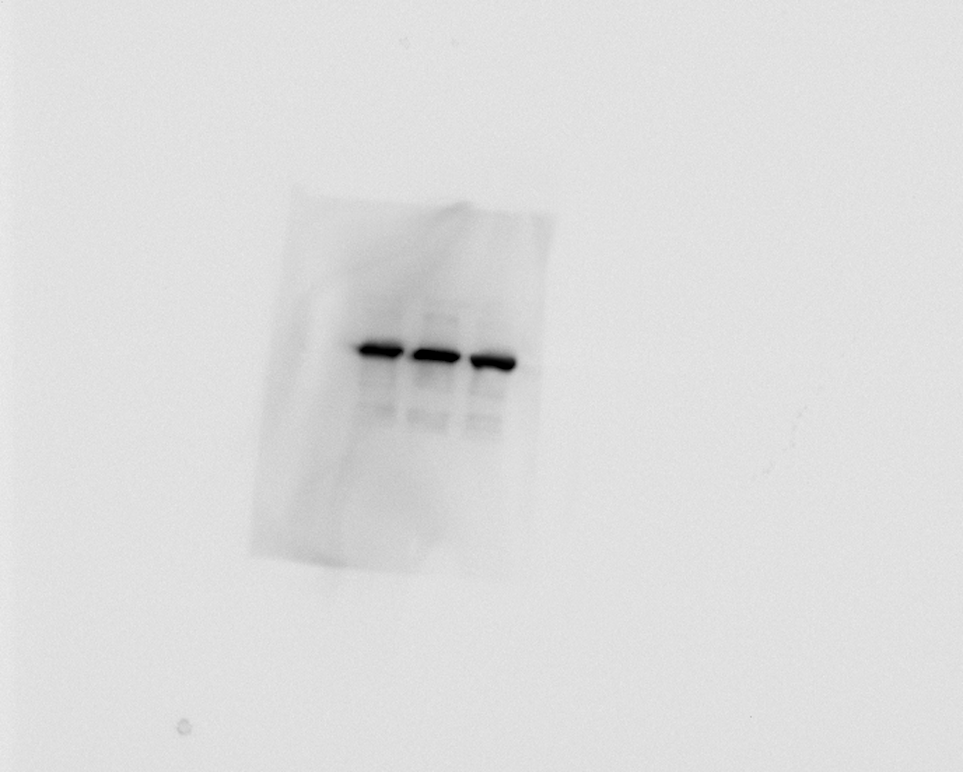** |
| **p-AKT** | **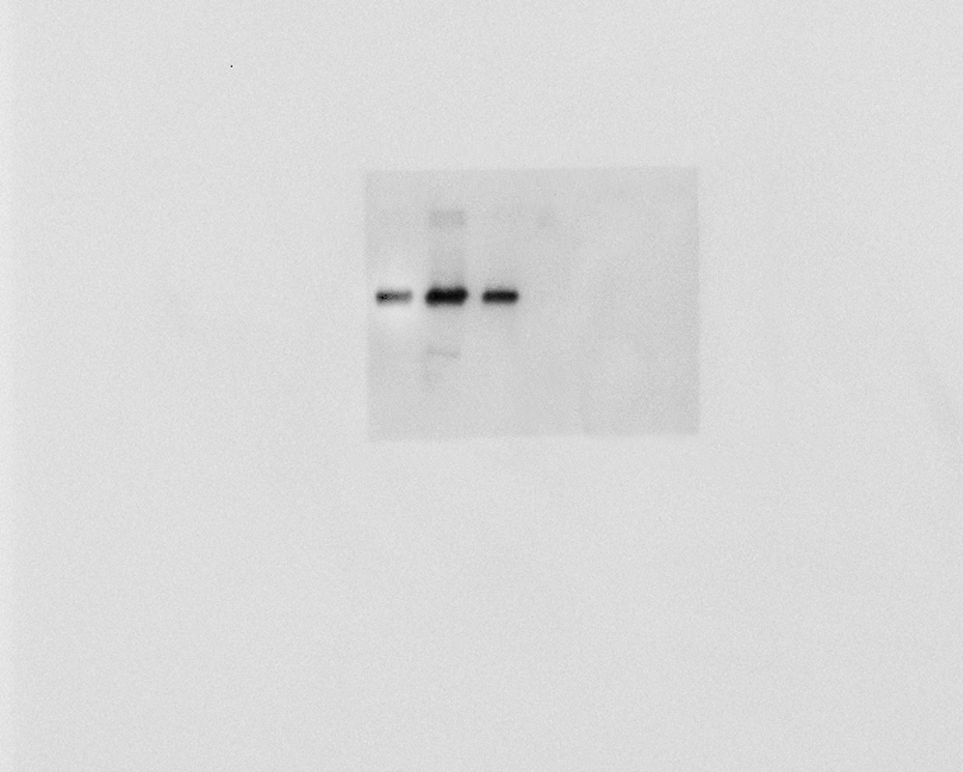** |
| **AKT** | **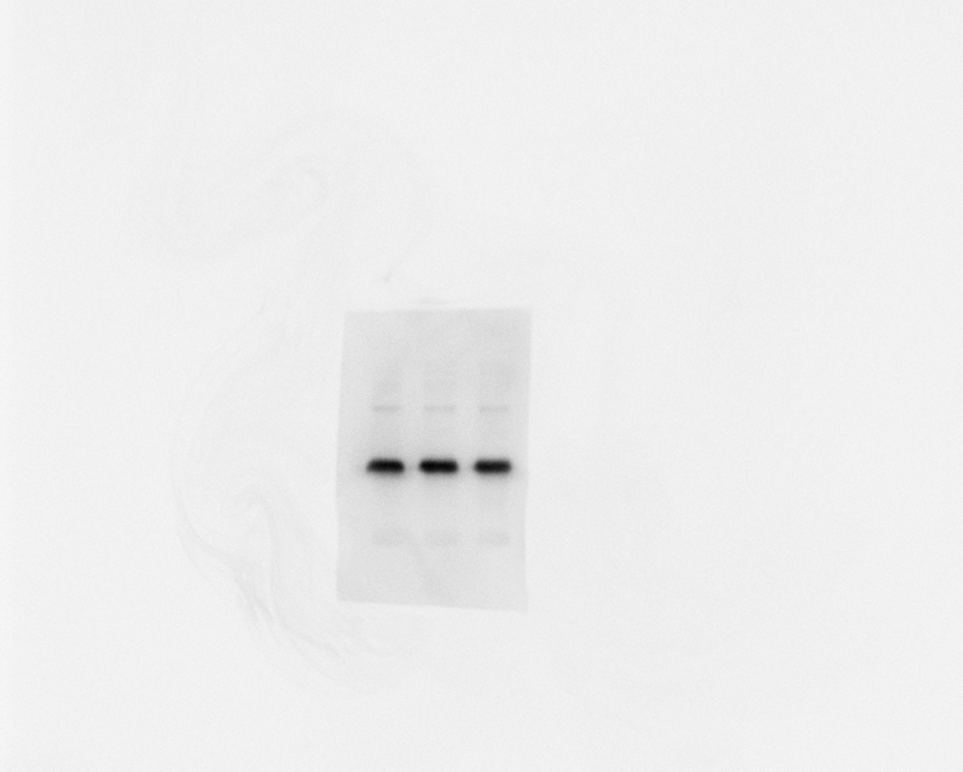** |
| **GAPDH** | **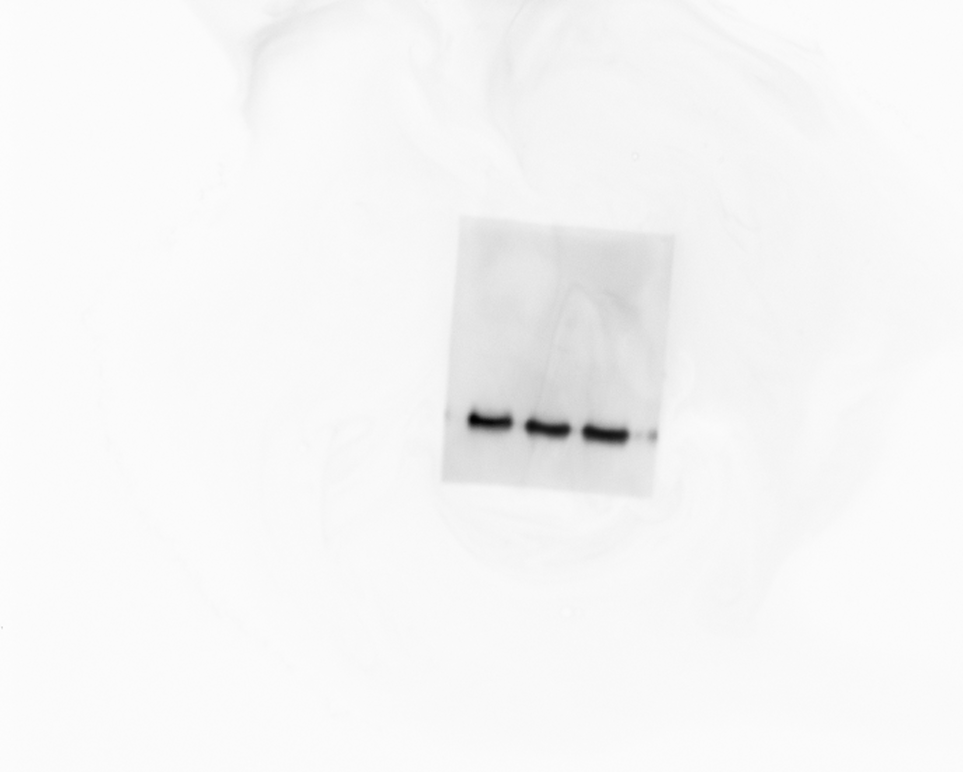** |
